# Typification and taxonomic status re-evaluation of 15 taxon names within the species complex *Cymbella affinis*/*tumidula*/*turgidula* (Cymbellaceae, Bacillariophyta)

**DOI:** 10.3897/phytokeys.53.4782

**Published:** 2015-07-21

**Authors:** Weliton José da Silva, Regine Jahn, Thelma Alvim Veiga Ludwig, Friedel Hinz, Mariângela Menezes

**Affiliations:** 1 Labfico, Departamento de Botânica, Museu Nacional, Universidade Federal do Rio de Janeiro, Rio de Janeiro, Brazil; 2 Programa de Pós-graduação em Biodiversidade Vegetal, Instituto de Ciências Biológicas, Universidade Federal de Goiás, Goiânia, Brazil; 3 Botanischer Garten und Botanisches Museum Berlin-Dahlem, Freie Universität Berlin, Berlin, Germany; 4 Departamento de Botânica, Setor de Ciências Biológicas, Centro Politécnico, Universidade Federal do Paraná, Curitiba, Brazil; 5 Alfred-Wegener-Institut, Helmholtz-Zentrum für Polar- und Meeresforschung, Bremerhaven, Germany

**Keywords:** Diatoms, typification, Cymbellales, *Cymbella tropica*, *Cymbella subturgidula*, *Cymbella rheophyla*, *Cymbella uenoi*

## Abstract

Specimens belonging to the *Cymbella affinis* / *Cymbella tumidula* / *Cymbella turgidula* species complex have many taxonomic problems, due to their high morphological variability and lack of type designations. Fifteen taxon names of this complex, distributed in five species, were re-evaluated concerning their taxonomic status, and lectotypified based on original material. In addition to light microscopy, some material was analyzed by electron microscopy. Four new combinations are proposed in order to reposition infraspecific taxa.

## Introduction

The history of the genus *Cymbella* C.Agardh is replete with taxonomic complexities. Within these complexes many species are similar in valve morphology. Much of the confusion in these complexes was caused by poor species descriptions including specimen images and the lack of designation of types, which has been required by the International Code of Nomenclature for algae, fungi, and plants (ICN) only since 1958 (McNeill et al. 2012).

In the most recent revision of the genus *Cymbella*, Krammer (2002) characterized and emended species descriptions within the complex *Cymbella affinis* Kütz. / *Cymbella tumidula* Grunow / *Cymbella turgidula* Grunow. This has generated confusion in the taxonomy of the group, specifically concerning the typification of *Cymbella affinis*. The concept of *Cymbella affinis* as proposed by Krammer (2002) involved the synonymization of *Cymbella affinis* and *Cymbella tumidula* Grunow, and the restoration of *Cymbella excisa* Kütz. *Cymbella excisa* has previously been treated by some authors as a synonym of *Cymbella affinis*, at the same or at an infraspecific rank (e.g. Cleve 1894, Grunow 1882, Patrick and Reimer 1975). According to Krammer (2002), the specimens treated as *Cymbella affinis* have cymbelloid outlines, with the axial area straight to slightly curved and a prominent central area. In these specimens the valvar ends are more protracted and more densely striated and areolated than in *Cymbella excisa*, which has the axial area curved and an indistinct central area. Moreover, *Cymbella excisa* commonly has an excision in the ventral middle part of the valve.

Krammer in Krammer and Lange-Bertalot (1986) also designated a neotype for *Cymbella turgidula*, a species with an outline very similar to *Cymbella affinis* but with wider, fewer and uniformly distributed striae, and less-dense punctae in the striae. In the same contribution, Krammer also described the new species *Cymbella subturgidula* Krammer, which he distinguished from *Cymbella turgidula* by its narrower breadth, higher length/breadth ratio, and the size and shape of the central area.

Tuji (2007) designated a lectotype for *Cymbella affinis* from an original illustration provided by Kützing (1844, Pl. 6, Fig. 15). Moreover, he assigned an epitype to a single specimen in preparation BM 18530 (Tuji 2007, Fig. 9) which was made from the original sample, which is Kützing’s packet 333.

Tuji (2007) observed that the designation of a neotype for *Cymbella turgidula* by Krammer in Krammer and Lange-Bertalot (1986) was inappropriate. This designation was made because Krammer was unable to locate the original samples or preparations (Krammer and Lange-Bertalot 1986). Tuji (2007) found the original slide (syntype) used by Grunow in Schmidt (1875) to describe *Cymbella turgidula*, and designated an original illustration as a lectotype and one specimen on Grunow’s slide as an epitype.

The material of *Cymbella uenoi* Skvortsov was also revisited by Tuji (2007), which according to him would make *Cymbella subturgidula* a synonym. He also proposed the new combination Cymbella uenoi f. nipponica (Skvortsov) Tuji for Cymbella turgidula var. nipponica Skvortsov [≡ *Cymbella rheophila* Ohtsuka].

Despite the changes, the revision of Tuji (2007) did not resolve the status of taxa such as *Cymbella tumidula* or *Cymbella excisa*. Moreover, he overlooked some requirements by the ICN with respect to the validity of the name *Cymbella uenoi*.

The aim of the present study was to elucidate the current taxonomic status of *Cymbella tumidula*, *Cymbella excisa* and *Cymbella subturgidula*, as well as of *Cymbella uenoi* and Cymbella uenoi f. nipponica [≡ Cymbella turgidula var. nipponica; ≡ *Cymbella rheophila*]; to revisit the infraspecific taxa encompassed in this complex of species; and to lectotypify the ambiguous taxa.

## Materials and methods

We analyzed the protologues and morphological features from materials of Cymbella affinis var. affinis, Cymbella affinis var. procera, Cymbella excisa var. excisa, Cymbella excisa var. procera, Cymbella excisa var. angusta, Cymbella excisa var. subcapitata, Cymbella tumidula var. tumidula, *Cymbella salinarum*, *Cymbella turgida*, *Cymbella tropica*, *Cymbella subturgidula*, Cymbella turgidula var. nipponica, *Cymbella reophila*, Cymbella uenoi f. uenoi, and Cymbella uenoi f. nipponica (Table 1). The characterization of *Cymbella salinarum* and Cymbella uenoi f. uenoi was based on the illustration of the holotype provided by Krammer (2002, Fig. 25: 13) and of the epitypes illustrated by Tuji (2007, Figs 14–19).

**Table 1.** Metric features of the material of the complex *Cymbella affinis*/*tumidula*/*turgidula* analyzed in this study (*n*= 29–34 individuals).

Taxon (published name)	Current name	Material	Length (µm)	Breadth (µm)	L/B ratio	Striae in middle part (in 10 µm)	Striae close to the ends (in 10 µm)	Puncta (in 10 µm)	Stigmata
*Cymbella affinis* Kütz. sensu Krammer (2002)	Cymbella tumidula Grunow var. tumidula	Preparation 1198F IOK in BRM, from France, Normandy, Falaise, coll. Brébisson, in BRM (Figs 50–56)	25.4–29.8	7.8–8.7	3.4–3.9	10–15	12–19	26–33	2–3
*Cymbella affinis* Kützing (1844)	Cymbella affinis Kützing var. affinis	Lectotype designated by Tuji (2007), preparation BM 18530, from France, Falaise, coll. Brébisson, in BM (Figs 1–7)	22.5–26.7	7.0–8.5	2.9–3.4	9–12	11–18	19–27	1
Cymbella affinis var. procera Krammer (2002)	Cymbella tumidula var. procera (Krammer) W.Silva, comb. nov.	Holotype, preparation 714 IOK, from Serbia, Rogatica, 1976, in BRM (Figs 57–63)	27.0–40.3	9.0–10.2	2.9–4.2	9–13	13–18	25–31	2–5
Cymbella excisa var. procera Krammer (2002)	Cymbella affinis var. neoprocera W.Silva, comb. nov. et nom. nov.	Holotype, preparation 212A IOK, from West Germany, Eifel, Totenmaar, 22.2.1974, in BRM (Figs 22–28)	21.5–41.0	8.0–11.0	2.7–4.0	9–13	11–14	22–27	1
*Cymbella excisa* Kützing (1844)	Cymbella affinis var. excisa (Kütz.) Grunow	Isolectotype, preparation 1131G IOK, Hauck No. 72 from Italy, 26.3.1837, in BRM (Figs 15–21)	23.1–28.5	7.0–9.5	2.7–3.9	9–12	11–17	24–26	1
Cymbella excisa var. angusta Krammer (2002)	Cymbella affinis var. angusta (Krammer) W.Silva, comb. nov.	Holotype, preparation 752 IOK, from Croatia, Lake Gavanovac, 18.5.1976, in BRM (Figs 29–35)	20.5–35.0	6.0–8.5	3.2–4.6	9–15	11–18	19–29	1
*Cymbella excisa* Kützing (1844)	Cymbella affinis var. excisa (Kütz.) Grunow	Lectotype (designated here), preparation BM 18543, from Hauk No. 72 from Italy, 26.3.1837, in BM (Figs 8–14)	21.5–26.5	7.0–8.7	2.6–3.5	9–13	11–16	21–25	1
Cymbella excisa var. subcapitata Krammer (2002)	Cymbella affinis var. subcapitata (Krammer) W.Silva, comb. nov.	Holotype, preparation 717A IOK, from Hungary, Balaton, 6.77, in BRM (Figs 36–42)	21.0–31.7	7.4–9.0	2.7–4.0	8–12	9–15	26–31	1
*Cymbella subturgidula* Krammer (2002)	*Cymbella subturgidula* Krammer	Lectotype, preparation 1046E IOK, from Korea, Ulchin County, Kyungsang Pukdo, Kwangchun River, in BRM (Figs 71–77)	30.3–37.4	9.0–12.8	2.6–3.5	9–11	12–15	21–24	2
*Cymbella tropica* Krammer (2002)	*Cymbella tropica* Krammer	Holotype, preparation 1015D IOK, from Venezuela, Rio Manizanes, coll. Rumrich 4.4.1990, in BRM (Figs 64–70)	34.5–42.7	10.0–12.0	3.1–4.0	9–12	11–12	21–24	1
*Cymbella tumidula* Grunow in Schmidt (1875)	Cymbella tumidula Grunow var. tumidula	Lectotype (designated here); Epitype (designated here), preparation BM 18543, from Hauck No. 72, from Italy, 26.3.1837, in BM (Figs 43–49)	26.8–34.7	7.8–8.7	3.4–4.0	10–15	12–19	26–33	2–4
*Cymbella salinarum* Grunow in Schmidt (1875)	Cymbella tumidula var. salinarum (Grunow) Cleve	Holotype illustration provided Krammer (2002, Fig. 25: 13)	34.0	10.7	3.2	14	15	–	1
*Cymbella turgidula* Grunow in Schmidt (1875)	*Cymbella turgidula* Grunow	Epitype designated by Tuji (2007), preparation 1504, from Puerto Rico, in the Grunow Collection, in W	36.5–45.0	10.8–13.3	3.2–3.4	9–12	11–17	21–24	1–3
Cymbella turgidula var. nipponica Skvortsov (1936) [≡ *Cymbella rheophila* Ohtsuka in Ohtsuka and Tuji (2002); ≡ Cymbella uenoi f. nipponica (Skvortsov) Tuji (2007)]	*Cymbella subturgidula* Krammer	Isolectotype, preparation R 214.928, from sample 0983 from Japan, Lake Biwa, coll. Tamiji Kawamura, 03.11.1915, in R (Figs 78–80)	28.1–39.0	10.0–13.2	2.8–3.0	9–12	12–15	21–26	1–2
		Preparations R 214.929 and R 214.930, from sample 1062, from Japan, Yamanoshita Bay in Lake Biwa, Otsu City, Shiga Prefecture, coll. Yasuko Iwao in 23.01.1993, in R (Fig. 81)	27.7–39.2	10.3–13.1	2.5–3.6	9–12	12–15	22–26	1–2
		Preparations R 214.931 and R214.932, from from sample 1093, from Japan, cobble in Lake Biwa, at Uchidehama, Otsu City, Shiga Prefecture, coll.Yasuko Iwao, 03.03.1993, in R (Figs 82–84)	26.5–40.1	11.1–13.5	2.3–3.4	9–13	12–14	21–24	2–3
*Cymbella uenoi* Skvortsov *ex*Tuji (2007)		Epitype and isotype illustrations provided Tuji (2007, Figs 21–24, 26)	26.3–41.0	9.0–12.7	2.9–3.2	9–11	12-15	21–24	2

Materials of *Cymbella affinis* (BM18530), *Cymbella excisa* (BM 18543), *Cymbella tumidula* (BM 18543), Cymbella turgidula var. nipponica (R 214.928, R 214.929, R 214.930, R 214.931 and R 214.932) were analyzed in the Laboratório de Ficologia, Museu Nacional, Rio de Janeiro, Brazil, using an Olympus BX 51 microscope (Olympus, Tokyo, Japan) fitted with an Olympus Q-Color digital camera. Images were processed with Q capture Pro QImaging© software.

Materials of *Cymbella affinis* sensu Krammer (1198F IOK BRM) and Cymbella affinis var. procera (714 IOK BRM) were examined in the laboratory of the Botanischer Garten und Botanisches Museum (BGBM), Berlin, Germany, using a Zeiss Axio Imager 4.2 microscope (Carl Zeiss MicroImaging GmbH, Berlin), and the images were captured through an MRc/MRm system (Carl Zeiss MicroImaging) and the software AxioVision Rel. 4.8 (Carl Zeiss MicroImaging).

We also analyzed materials *Cymbella excisa* sensu Krammer (1131G IOK BRM), Cymbella excisa var. procera (212A IOK BRM), Cymbella excisa var. angusta (752 IOK BRM), Cymbella excisa var. subcapitata (717A IOK BRM), *Cymbella tropica* (1015D IOK BRM) and *Cymbella subturgidula* (1046E IOK BRM) with a Zeiss Axioplan microscope (Carl Zeiss, Jena, Göttingen, Germany) with an Olympus XC50 capture system (Olympus, Tokyo, Japan) and the software analySIS Image Processing (Soft Imaging System, Münster, Germany), at the Alfred-Wegener-Institut für Polar- und Meeresforschung (BRM), Bremerhaven, Germany.

Scanning Electron Microscopy (SEM) analyses were carried out only for Cymbella turgidula var. nipponica. Samples were deposited on cover slips and attached to aluminum stubs using LeitSilver® (Sigma-Aldrich, Berlin, Germany). The material was coated with 150–200 Å of gold in an Emitech K 550 sputter coater (Quorum Technologies Ltd., Kent, UK). The material was analyzed in a Jeol JSM-6390 scanning electron microscope (Jeol, USA), operated at 6–8 kV, spot size 10–30, in the electron microscopy laboratory in the Museu Nacional, Rio de Janeiro, Brazil.

The term “degree of dorsiventrality” is used here to define how dissimilar the sides of the valvae are on the apical axis. The symbols “≡”, “=” and “–” preceding specific and infraspecific names are used to represent homotypic or nomenclatural, heterotypic or taxonomic, and concept synonyms, respectively, as used in the ICN (McNeill et al. 2012).

## Results

### Cymbella affinis

Taxon classificationPlantaeCymbellalesCymbellaceae

Kütz

Figs 1–7

Cymbella affinis Kütz., Bacill., 80, Pl. 6 Fig. 15, 1844.

#### Lectotype.

3^rd^ figure from the left in Kützing (1844, pl. 6, Fig. 15) designated by Tuji (2007).

#### Type locality.

“Falaise (France): De Brébisson! Herb. Binder., Schleswig: Herb. Binder.!”

#### Epitype.

An individual on preparation BM18530, from Kützing packet 333, in the Natural History Museum (BM), designated by Tuji (2007, Fig. 9).

#### Epitype locality.

Falaise, France, coll. De Brébisson.

Valvae lanceolate, dorsiventral, dorsal and ventral margins convex; ends barely protracted, rounded, to slightly subrostrate or subcapitate; length 22.5–26.5 µm, breadth 7.0–8.5 µm, L/B ratio 2.9–3.4; axial area narrow, linear-arched, indistinct central area; striae 9–12 in 10 µm, becoming 11–18 towards ends, one isolated pore at end of central striae on ventral side; 19–27 punctae in 10 µm.

#### Remarks.

Although Krammer (2002, p. 41) used material sampled by the same collector and from the same locality and related his new preparation to the type locality, his effort did not constitute a typification of *Cymbella affinis* as ruled by the ICN (Art. 7.10, McNeill et al. 2012). On the other hand, Tuji (2007) was the first to consider the original material of this taxon and performed a lectotypification and epitypification, and as such, must be followed according to Articles 9.19 and 9.20 (McNeill et al. 2012).

The lectotype valve of *Cymbella affinis* is similar to the lectotype valve of *Cymbella excisa*, except for the excision in the middle part of the valve present in the majority of specimens (Figs 8–21). Populations of this complex from different parts of the world may or may not have excisions, but this character is present in the majority of specimens from the populations examined (Krammer 2002). Therefore, we consider *Cymbella excisa* and *Cymbella affinis* as belonging to the same species with differences at the varietal rank based on phenotypic expression and ecological modifications. Genetic studies are still to be completed.

**Figures 1–28.**
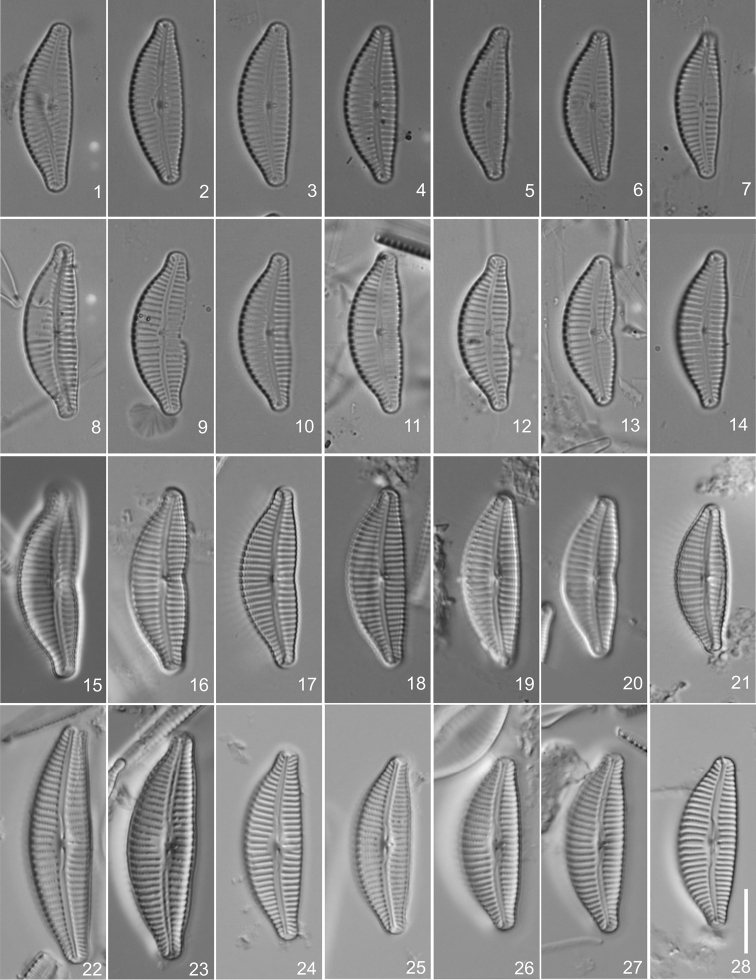
*Cymbella affinis* species complex **1–7** Cymbella affinis Kütz. var. affinis **8–14** Cymbella affinis var. excisa (Kütz.) Grunow, specimens from Trieste in preparation BM 18543 **11** Lectotype, designated here **15–21** Cymbella affinis var. excisa [– *Cymbella excisa* sensu Krammer], specimens from Trieste in preparation 1131 IOK **16** Isolectotype, designated here **22–28** Cymbella affinis var. neoprocera W.Silva, specimens from Germany in preparation 212A IOK, holotype **22** Lectotype, designated here. Scale bar: 10 µm.

The two taxa were both proposed by Kützing (1844, p. 80) and therefore have the same priority. In similar cases, Article 11.5 of the ICN rules that “the first such choice to be effectively published establishes the priority of the chosen name”.

Grunow (1882) proposed the new combination and the new status of *Cymbella excisa* for Cymbella affinis var. excisa (Kütz.) Grunow. This was the first publication that defined the priority of the epithet *affinis* over *excisa* at the specific level. Therefore, the name *Cymbella affinis* must be considered to be the name of the species when *Cymbella excisa* and *Cymbella affinis* are considered to be the same species, in conformity with Article 11.5 of the ICN (McNeill et al. 2012).

*Cymbella affinis* valves sensu Patrick and Reimer (1975, p. 57) have similar outlines as *Cymbella affinis* in the type population, but higher range of length and breadth values (length: 20–50 vs. 22.5–26.5 µm; breadth: 7–12 vs. 7.0–8.3 µm, respectively). However, these authors (Patrick and Reimer 1975), included representatives of different localities that could encompass different varieties subscribed to this taxon. The density of striae in the material from USA was similar in the middle part of the valve compared to the type material (9–11 vs. 9–12 striae in 10 µm, respectively) and lower when comparing them close to the ends of the valvae (12–14 vs. 11–18 striae in 10 µm, respectively) (Patrick and Reimer 1975).

Cymbella excisa var. procera Krammer (Figs 22–28), Cymbella excisa var. angusta Krammer (Figs 39–35) and Cymbella excisa var. subcapitata Krammer (Figs 36–42) also appear to be conspecific with *Cymbella affinis*, but the types show slight differences in their outlines and metric characteristics (Table 1). Thus, all these taxa are here transferred to *Cymbella affinis* using their respective infraspecific epithet.

### Cymbella affinis var. excisa

Taxon classificationPlantaeCymbellalesCymbellaceae

(Kütz.) Grunow

Figs 8–21

Cymbella affinis var. excisa (Kütz.) Grunow, Beitr. Paläont. Österreich.-Ungarns Orients, 2: 142, Pl. 19(1), Fig. 26, 1882.

#### Basionym.

*Cymbella excisa* Kütz., Bacill., 80, Pl. 6, Fig. 17, 1844.

#### Lectotype

**(designated here).** An individual marked with blue ring on preparation BM 18543, from Hauck No. 72, 26.4.1837, in the Natural History Museum (BM), London, United Kingdom, represented by Fig. 11.

#### Isolectotype

**(designated here).** An individual on preparation 1131G IOK, in the Alfred-Wegener-Institut für Polar- und Meeresforschung (BRM), Bremerhaven, Germany, represented by Fig. 16.

#### Type locality.

“Unter Oscillatorien in Bächen bei Triest”, Italy, 26.4.1837.

Valvae dorsiventral, dorsal margin broadly convex, ventral margin straight, usually with an excision in middle portion; ends subrostrate to rostrate; length 21.5–28.5 µm, breadth 7.0–9.5 µm, L/B ratio 2.6–3.9; axial area narrow, linear-arched, central area indistinct; striae 9–13 in 10 µm, becoming 11–17 toward ends, one isolated pore at end of central striae on ventral side; 21–26 punctae in 10 µm.

#### Remarks.

Similarly to *Cymbella affinis*, the lectotypification of *Cymbella excisa* designated by Krammer (2002, p. 26) cannot be considered according to Article 7.10 of the ICN (McNeill et al. 2012), because the phrase “designated here” or equivalent is required from 1 January 2001 and it was not included by Krammer (2002). Therefore, the lectotype designated by us cannot be considered a replacement of Krammer’s (2002) “lectotype”; rather, it is the first lectotypification of this taxon.

The main difference between Cymbella affinis var. excisa and the nominate variety is the presence of an excision in the middle portion of the ventral side of the valve, a characteristic common to populations of this taxon around the world (Krammer 2002). Therefore, we consider that the presence of excisions in all populations, not present in the type material of *Cymbella affinis*, constitutes sufficient grounds to consider Cymbella affinis var. excisa different from the nominate variety, which conforms to the statement by Grunow (Schmidt 1875); and not a different species.

Krammer (2002) recorded populations in the isotype material of Cymbella affinis var. excisa with length 17–41 µm, and breadth 6.0–10.7 µm, which were higher than populations in the lectotype material. Krammer (2002) was able to observe initial and post initial cells which were similar to the minimum and maximum length and breadth of this taxon. However, Krammer (2002) included specimens of the variety *excisa* and the nominate variety in his description.

### Cymbella affinis var. neoprocera

Taxon classificationPlantaeCymbellalesCymbellaceae

W.Silva, comb. nov. et nom. nov.

Figs 22–28

#### Basionym.

Cymbella excisa var. procera Krammer, Diatoms Europe 3: 159, Figs 9:1–7, 2002 (Figs 22–28).

#### Holotype.

Preparation 212A IOK, in the Alfred-Wegener-Institut für Polar- und Meeresforschung (BRM), Bremerhaven, Germany.

#### Lectotype

**(designated here).** An individual on preparation 212A IOK, in the Alfred-Wegener-Institut für Polar- und Meeresforschung (BRM), Bremerhaven, Germany; represented by Fig. 22.

#### Type locality.

Germany, Eifel, Totenmaar, 22.2.1974.

Valvae dorsiventral, dorsal margin broadly convex, ventral margin straight; ends not protracted, rounded, or subrostrate to rostrate; length 21.5–41.0 µm, breadth 8.0–11.0 µm, L/B ratio 2.7–4.0; axial area narrow, linear-arched, indistinct central area; striae 9–13 in 10 µm, becoming 11–14 toward ends, one isolated pore at end of central striae on ventral side; 22–27 punctae in 10 µm.

#### Remarks.

The combination of Cymbella excisa var. procera with *Cymbella affinis* would be illegitimate unless it was given a new name, because it would be a later homonym of Cymbella affinis var. procera Krammer. Specimens designated by Krammer (2002), i.e., the preparation 212A IOK (BRM), were found to belong to more than one taxon therefore to clarify the taxonomy we designated a lectotype as established in Art. 9.11 of the ICN (McNeill et al. 2012).

A broader range of metric characteristics were highlighted in this study compared to the characterization of the type population (Krammer 2002, p. 28). We find smaller (21.5–41.0 vs. 24–41 µm) and narrower (8.0–11.0 vs. 8.4–11.0 µm) representatives of this taxon compared to the Krammer’s (2002) records. Still considering Krammer’s (2002) findings, we observe more densely striated (9–13 vs. 9–11 striae in 10 µm) and less punctate (22–27 vs. 24–27 punctae in 10 µm) valves.

### Cymbella affinis var. angusta

Taxon classificationPlantaeCymbellalesCymbellaceae

(Krammer) W.Silva comb. nov.

Figs 29–35

#### Basionym.

Cymbella excisa var. angusta Krammer, Diatoms Europe 3: 159, Figs 9: 8–13, 2002.

#### Holotype.

Preparation 752 IOK, in the Alfred-Wegener-Institut für Polar- und Meeresforschung (BRM), Bremerhaven, Germany.

#### Lectotype

**Figures 29–42.**
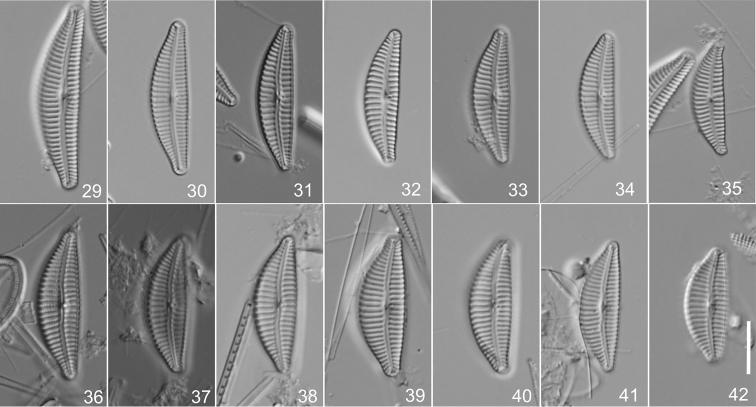
*Cymbella affinis* species complex **29–35** Cymbella affinis var. angusta (Krammer) W.Silva, specimens from Croatia in preparation 752 IOK, holotype **29** Lectotype, designated here **36–42** Cymbella affinis var. subcapitata (Krammer) W.Silva, specimens from Hungary in preparation 717 IOK, holotype **39** Lectotype, designated here. Scale bar: 10 µm.

**(designated here).** An individual on preparation 752 IOK, in the Alfred-Wegener-Institut für Polar- und Meeresforschung (BRM), Bremerhaven, Germany, represented by Fig. 29.

#### Type locality.

Croatia, watercourse near Lake Gavanovac, Plitvice.

Valves dorsiventral, dorsal margin broadly convex, ventral margin straight, usually with excision in middle portion; ends not protracted to slightly protracted, rounded, subrostrate or subcapitate; length 20.5–35.0 µm, breadth 6.0–8.5 µm, L/B ratio 3.2–4.6; axial area linear-arched, indistinct central area; striae 9–15 in 10 µm, becoming 11–18 towards ends, one isolated pore at end of central striae of ventral side; 19–29 punctae in 10 µm.

#### Remarks.

The material recorded here presented slight differences in the valve metrics compared to Krammer’s (2002, p. 28) characterization from the same preparation; differences included valve length (20.5–35.0 vs. 17.0–34.0 µm), breadth (6.0–8.4 vs. 6.7–8.2 µm), striae (9–15 vs. 11–14 in 10 µm) and number of punctae (19–29 vs. 25–28 in 10 µm).

Similar to Cymbella affinis var. neoprocera, specimens designated by Krammer (2002), i.e., the preparation 752 IOK (BRM), were found to belong to more than one taxon. Therefore we designated a lectotype for this taxon as established in Art. 9.11 of the ICN (McNeill et al. 2012).

### Cymbella affinis var. subcapitata

Taxon classificationPlantaeCymbellalesCymbellaceae

(Krammer) W.Silva comb. nov.

Figs 36–42

#### Basionym.

Cymbella excisa var. subcapitata Krammer, 2002, Diatoms of Europe 3: 159, Figs 10: 14–18.

#### Holotype.

Preparation 717A IOK, in the Alfred-Wegener-Institut für Polar- und Meeresforschung (BRM), Bremerhaven, Germany.

#### Lectotype

**(designated here).** An individual on preparation 717A IOK, in the Alfred-Wegener-Institut für Polar- und Meeresforschung (BRM), Bremerhaven, Germany, represented by Fig. 39.

#### Type locality.

Hungary, Balaton.

Valves dorsiventral, dorsal margin broadly convex, ventral margin straight, usually with excision in middle portion; ends barely protracted, rounded, to broadly protracted, subcapitate; length 21.0–31.7 µm, breadth 7.4–9.0 µm, L/B ratio 2.7–4.0; axial area linear-arched, indistinct central area; striae 8–12 in 10 µm, becoming 9–15 towards ends, one isolated pore at end of central striae of ventral side; 26–31 punctae in 10 µm.

#### Remarks.

Krammer (2002) distinguished this variety from the variety *excisa* based on the shape of the ends of the valvae. However, he (Krammer 2002, p. 28) did not provide metric characterizations of this the variety *subcapitata*, which we observed to agree with the characterizations of *Cymbella excisa* sensu Krammer (2002), except for the density of striae that was slightly lower in the variety *subcapitata*.

Specimens designated by Krammer (2002), i.e., the preparation 717A IOK (BRM), were found to belong to more than one taxon. Therefore we designated a lectotype as established in Art. 9.11 of the ICN (McNeill et al. 2012).

### Cymbella tumidula Grunow var. tumidula

Taxon classificationPlantaeCymbellalesCymbellaceae

Figs 43–56

Cymbella tumidula Grunow var. tumidula , in Schmidt, A. Schmidt’s Atlas Diatom.-Kunde, Pl. 9, Fig. 33, 1875.

#### Synonym.

*Cymbella affinis* Kütz. *sensu* Krammer, Diatoms Europe 3: 41, Figs 21:2–21, 22:1–20, 23:1–18, 2002. (Figs 50–56).

**Figures 43–63.**
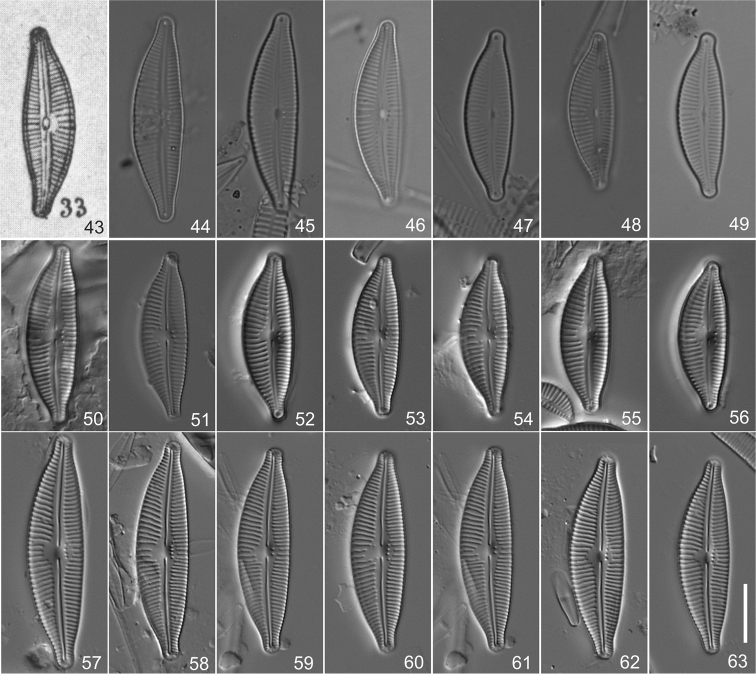
*Cymbella tumidula* species complex **43** Original illustration provided by Grunow in Schmidt (1875, Pl. 9, Fig. 33), lectotype, here designated **44–49** Cymbella tumidula var. tumidula, specimens from Trieste in preparation BM 18543 **49** Epitype, here designated **50–56** Cymbella tumidula var. tumidula [*Cymbella affinis* sensu Krammer (2002)], specimens from Trieste in preparation 1131G IOK **54** Isolectotype, designated here **57–63** Cymbella tumidula var. procera (Krammer) W.Silva, specimens from Serbia in preparation 714 IOK, holotype **57** Lectotype, designated here. Scale bar: 10 µm.

#### Lectotype

**(designated here).** Plate 9, Fig. 33 from Schmidt (1875a) (Fig. 43).

#### Type locality.

Italy, Trieste, 26.4.1837.

#### Epitype

**(designated here).** An individual marked with a red ring on preparation BM 18543 from Hauck No. 72, 26.4.1837, in the Natural History Museum (BM), London, United Kingdom, represented by Fig. 49.

#### Isoepitype

**(designated here).** An individual on preparation 1131G IOK, from Hauck No. 72, 26.4.1837, in the Alfred-Wegener-Institut für Polar- und Meeresforschung (BRM), Bremerhaven, Germany, represented by Fig. 54.

#### Epitype locality.

Italy, Trieste, 26.4.1837.

Valves lanceolate, dorsiventral, dorsal and ventral margins convex; subcapitate ends; length 25.4–34.7 µm, breadth 7.8–8.7 µm, L/B ratio 3.4–4.0; axial area linear-lanceolate, straight to arched, central area irregular to rounded; striae 10–15 in 10 µm, becoming 12–19 toward ends, 2–4 isolated pores at end of central striae on ventral side; 26–33 punctae in 10 µm.

#### Remarks.

The lectotypification of *Cymbella affinis* allowed us to consider *Cymbella affinis* and *Cymbella tumidula* [– *Cymbella affinis* sensu Krammer], lectotypified and epitypified here, as independent species. *Cymbella tumidula* has a more lanceolate outline, subcapitate ends, and a lower degree of dorsiventrality than *Cymbella affinis*. The striae in the middle part of the valve are shorter and unevenly distributed in *Cymbella tumidula*, forming a distinct central area (Figs 43–63), in contrast to *Cymbella affinis* where the central area is indistinct and the striae are uniformly distributed. In addition, all specimens of the type material of *Cymbella affinis* have only one stigma, whereas in *Cymbella tumidula* 1–5 stigmata can be observed.

Specimens from the population of the holotype material of Cymbella affinis var. procera were very similar in outline but larger and wider than Cymbella tumidula var. tumidula, resulting in higher maximum length/breadth ratios. However, all metric characteristics of Cymbella affinis var. procera intergraded with Cymbella tumidula var. tumidula, and therefore this taxon was transferred to Cymbella tumidula var. procera.

The characteristics of *Cymbella affinis* sensu Krammer (2002, p. 41) were similar to the type population of *Cymbella tumidula*, but with a wider range of values. This includes length (17–34 vs. 25.4–34.7 µm, respectively) and breadth (7.5–9.5 vs. 7.8–8.7 µm). *Cymbella affinis* sensu Krammer (2002, p. 41) also presented narrower range of values of striae in 10 µm (10–13, becoming 13–15 toward ends vs. 10–15 in 10 µm, becoming 12–19 toward ends, respectively) and density of punctae (27–32 vs. 26–33 punctae in 10 µm) than *Cymbella tumidula*.

### Cymbella tumidula var. procera

Taxon classificationPlantaeCymbellalesCymbellaceae

(Krammer) W.Silva comb. nov.

Figs 57–63

#### Basionym.

Cymbella affinis var. procera Krammer, Diatoms Europe 3: 161, Figs 22: 8–13, 2002.

#### Holotype.

Preparation 714 IOK, in the Alfred-Wegener-Institut für Polar- und Meeresforschung (BRM), Bremerhaven, Germany.

#### Lectotype

**(designated here).** An individual on preparation 714 IOK, in the Alfred-Wegener-Institut für Polar- und Meeresforschung (BRM), Bremerhaven, Germany, represented by Fig. 57.

#### Type locality.

Serbia, Rogatica (abundant in chalk-rich spring), 1976.

Valves lanceolate, dorsiventral, dorsal and ventral margins convex; ends subrostrate or subcapitate; length 27.0–40.3 µm, breadth 9.0–10.2 µm, L/B ratio 2.9–4.2; axial area linear-lanceolate, slightly arched, central area rounded; striae 9–13 in 10 µm, becoming 13–18 toward ends, 2–5 isolated pores; 25–31 punctae in 10 µm.

#### Remarks.

According to Krammer (2002), this variety differs concerning wider valves (higher than 9.5 µm) from the nominate variety of *Cymbella affinis* sensu Krammer. We recorded specimens of Cymbella tumidula var. procera with 9 µm breadth, which were higher than the nominate variety (9.0–10.2 vs. 7.8–8.7 µm).

Specimen designated by Krammer (2002), i.e., the preparation 714 IOK (BRM), were found to belong to more than one taxon. Therefore we designated a lectotype as established in Art. 9.11 of the ICN (McNeill et al. 2012).

### Cymbella tumidula var. salinarum

Taxon classificationPlantaeCymbellalesCymbellaceae

Grunow

Cymbella tumidula var. salinarum (Grunow) Cleve, Kongl. Svenska Vetensk.-Akad. Handl., ser. 4, 26(2): 171, 1894.

#### Basionym.

*Cymbella salinarum* Grunow in Schmidt, A. Schmidt’s Atlas Diatom.-Kunde, Pl. 9, fig. 28, 1875.

#### Holotype.

Preparation 1603 in the Grunow Collection in the Naturhistorisches Museum Wien (W).

#### Lectotype

**(designated here).** An individual on preparation 1603, in the Grunow Collection in the Naturhistorisches Museum Wien (W), represented by the illustration in Krammer (2002, Fig. 25: 13).

#### Type locality.

Salinen von Zaule (Trieste, Italy).

Valves lanceolate, dorsiventral, dorsal and ventral margins convex; ends subcapitate; length 34.0 µm, breadth 10.7 µm, L/B ratio 3.2; axial area linear-lanceolate, slightly arched, central area rounded; striae 14 in 10 µm, becoming 15 toward ends, 1 isolated pore.

#### Remarks.

This taxon presents morphometric characteristics similar to Cymbella tumidula var. tumidula, except it has wider valves. Cleve (1894) recorded specimens of Cymbella tumidula var. salinarum with 27–40 µm length, 8–10 µm breadth, and 11 or 12 striae in 10 µm, and considered that the only difference between this taxon and Cymbella tumidula var. tumidula was the shape of the ends. Although in poor condition, in preparation 1603 we did not find differences between the shape of the valvar ends of the variety *salinarum* and the nominate variety. However, Cymbella tumidula var. salinarum has higher breadth values compared to the type population of Cymbella tumidula var. tumidula, even in populations of this taxon as recorded by Krammer (2002) from Falaise where initial and post initial cells were found. Moreover, the occurrence of Cymbella tumidula var. salinarum has been restricted to brackish waters.

Krammer (2002, Fig. 25: 13) provided the illustration of an individual of the type of *Cymbella salinarum*. The individual represented by him (Krammer 2000) was similar to Cymbella tumidula var. tumidula. However, it was larger and had only one isolated pore, differing from *Cymbella tumidula*, which has more than two isolated pores (Figs 50–56). Thus, in contrast to Krammer (2002), who treated *Cymbella salinarum* at the specific level, we consider this taxon at the infraspecific rank as did Cleve (1894).

### Cymbella turgidula

Taxon classificationPlantaeCymbellalesCymbellaceae

Grunow

Cymbella turgidula Grunow, in Schmidt, A. Schmidt’s Atlas Diatom.-Kunde, Pl. 9, Figs 23–26, 1875.

#### Lectotype.

Plate 9, Fig. 23 in Schmidt (1875), designated by Tuji (2007).

#### Type locality.

Puerto Rico and Kahyenmathay.

#### Epitype.

An individual on slide 1504 in the Grunow Collection in the Naturhistorisches Museum Wien (W), designated by Tuji (2007, Fig. 15).

#### Epitype locality.

Puerto Rico.

Valves lanceolate, dorsiventral, dorsal margins broadly convex and ventral margin convex; ends barely protracted, narrowly rounded, or subcapitate; length 36.5–45.0 µm, breadth 10.8–13.3 µm, L/B ratio 3.2–3.4; axial area linear, arched, central area indistinct to rounded; striae 9–12 in 10 µm, becoming 11–17 toward ends, 1–3 isolated pores; 21–24 punctae in 10 µm.

### Cymbella tropica

Taxon classificationPlantaeCymbellalesCymbellaceae

Krammer

Figs 64–70

Cymbella tropica Krammer, Diatoms Europe 3: 61, Figs 44:1–10, 49: 12, 13, 2002.

#### Holotype.

Preparation 1015D IOK, in the Alfred-Wegener-Institut für Polar- und Meeresforschung (BRM), Bremerhaven, Germany.

#### Lectotype

**Figures 64–70.**
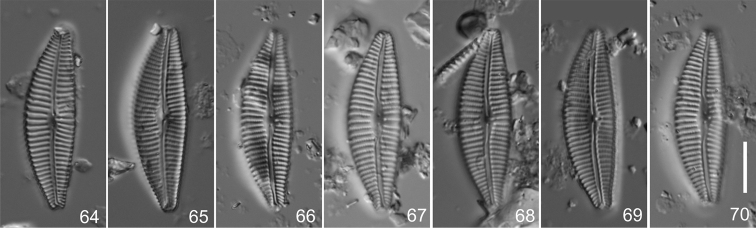
*Cymbella tropica* Krammer from Venezuela in preparation 1015D IOK, holotype **64** Lectotype, designated here. Scale bar: 10 µm.

**(designated here).** An individual on preparation 1015D IOK, in the Alfred-Wegener-Institut für Polar- und Meeresforschung (BRM), Bremerhaven, Germany, represented by Fig. 64.

#### Type locality.

Venezuela, Rio Manizanes, coll. Rumrich, 4.4.1990.

Valves lanceolate, dorsiventral, dorsal and ventral margins convex; ends barely protracted, rounded, or subcapitate, slightly deflected to ventral margin; length 34.5–42.7 µm, breadth 10.0–12.0 µm, L/B ratio 3.1–4.0; axial area linear, arched, central area indistinct to slightly rounded; striae 9–12 in 10 µm, becoming 11–12 toward ends, 1 isolated pore; 21–24 punctae in 10 µm.

#### Remarks.

Krammer (2002) described *Cymbella tropica* and recorded differences in the size, length/breadth ratio, and the presence of only one stigma as consistent diagnostic differences between this species and *Cymbella turgidula*. Tuji (2007) recorded the occurrence of specimens of *Cymbella turgidula* with 1–3 isolated pores, which was also observed by Krammer (2002) in his material. We observed a continuum between the metric characteristics of *Cymbella tropica* and *Cymbella turgidula*, even in those characters that were considered by Krammer (2002) as differentiating. However, the outline was more lanceolate, the ends more protracted and slightly deflected to the ventral side, and the degree of dorsiventrality was lower in *Cymbella tropica* compared to *Cymbella turgidula*.

### Cymbella subturgidula

Taxon classificationPlantaeCymbellalesCymbellaceae

Krammer

Figs 71–84 , 85–89 , 90–97

Cymbella subturgidula Krammer, Diatoms Europe 3: 166, Figs 44: 19–21, 2002.

#### Synonyms.

= Cymbella turgidula var. nipponica Skvortzov, Philipp. J. Sci. 61: 283, Figs 2:8, 4:4, 1936. (Figs 78–97)

– *Cymbella uenoi* Skvortsov in Skvortsov & Noda, Sci. Rep. Niigata Univ., ser. D (Biol.) 8: 19, Pl. 3, Fig. 3, 1971. (nom. inval.)

= *Cymbella rheophila* Ohtsuka in Ohtsuka & Tuji, Phycol. Res. 50: 245, Figs 7, 8, 2002.

= *Cymbella uenoi* Skvortsov *ex* Tuji, Diatom 23: 50, Figs 20–25, 2007.

= Cymbella uenoi f. nipponica (Skvortsov) Tuji, Diatom 23: 54, Fig. 26, 2007.

#### Holotype.

Preparation 1046E IOK [not “1046c IOK”], in the Alfred-Wegener-Institut für Polar- und Meeresforschung (BRM), Bremerhaven, Germany.

#### Lectotype

**(designated here).** An individual on preparation 1046e IOK, in the Alfred-Wegener-Institut für Polar- und Meeresforschung (BRM), Bremerhaven, Germany, represented by Fig. 73.

**Figures 71–84.**
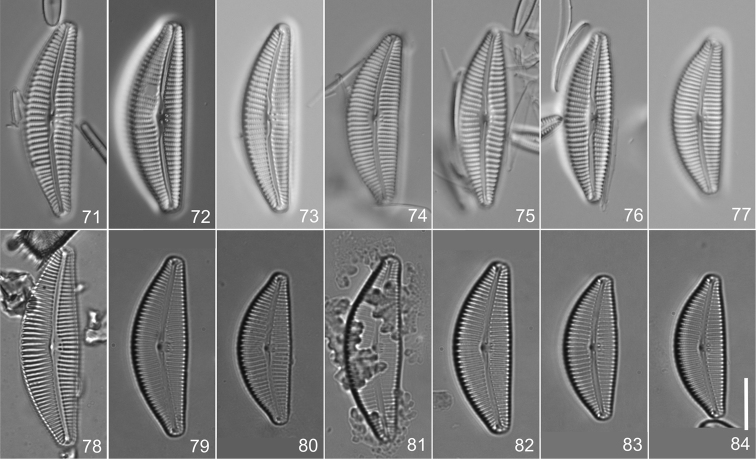
*Cymbella subturgidula* Krammer **71–77** Specimens from Korea in preparation 1046E IOK, holotype **73** Lectotype, designated here **78–80** Specimens from Japan in preparation R 214.928 **78**Isolectotype, designated here, of Cymbella turgidula var. nipponica Skvortsov [≡ *Cymbella rheophila* Ohtsuka], heterotypic synonym of *Cymbella subturgidula* **81** Specimen from Japan in preparation R 214.930 **82–84** Specimens from Japan in preparation R 214.932. Scale bar: 10 µm.

#### Type locality.

Korea, Ulchin County, Kyungsang Pukdo, Kwangchun River.

Valvae slightly lanceolate to lanceolate, dorsiventral, dorsal margin broadly convex and ventral margin straight to convex; ends barely protracted, subrostrate to broadly subcapitate; length 26.3–41.0 µm, breadth 9.0–13.5 µm, L/B ratio 2.3–3.6; axial area linear to linear-lanceolate, arched, central area indistinct to slightly rounded; striae 9–13 in 10 µm, becoming 12–15 toward ends, 1–3 isolated pores; 21–26 punctae in 10 µm. In SEM, the striae showed lineolate punctae externally and internally, the striae are composed by an alveolus internally, surrounded by thick costae; the isolated pores are rounded externally; internally, the alveoli of the isolated pores are irregularly obovate and connected to intercostae, the margins with tooth-like structures (brocca); one apical pore field (APF) not divided by the external terminal fissure of the raphe can be observed on each pole of the valvae; the terminal nodule extends to the dorsal side, under the APF and has a short branch that penetrates the APF apically; the helictoglossae lie under the terminal nodule and are deflected to the dorsal side.

#### Remarks.

Krammer (2002) described *Cymbella subturgidula* based on preparation 1046c, which he designated as the holotype. This preparation was sought in the Alfred-Wegener-Institut für Polar- und Meeresforschung (BRM), where the entire Krammer Collection was transferred. However, preparation 1046c IOK is from Argentina in South America, and not from the holotype designated from Korea. In the protologue of *Cymbella subturgidula*, Krammer (2002, p. 278 and 279) illustrated three specimens from preparation 1046E IOK, from Korea. Thus, the existence of slide 1046c IOK from Argentina, which is incongruent with the type locality, and the existence of slide 1046E which was used by Krammer to illustrate *Cymbella subturgidula*, led us to consider the indication of preparation 1046c IOK as a typographical error.

*Cymbella subturgidula* and *Cymbella turgidula* are closely related species. However, *Cymbella turgidula* is more lanceolate and has a higher degree of dorsiventrality than *Cymbella subturgidula*. Moreover, *Cymbella turgidula* is slightly broader than *Cymbella subturgidula*, with a more prominent ventral side of the valve. The ends in the two species are different, being subrostrate-rounded in *Cymbella turgidula* and slightly subrostrate-truncate in *Cymbella subturgidula*. The central area is more distinct in *Cymbella turgidula* than *Cymbella subturgidula*. Although the number of punctae in 10 µm is the same in both species, the striae in *Cymbella subturgidula* seem to be more coarsely punctuated than in *Cymbella turgidula*.

Cymbella turgidula var. nipponica was described by Skvortzow (1936). He considered that this taxon differed from the nominate variety due to the elongated valve, slightly undulate ventral margin, and broad rostrate ends. Ohtsuka and Tuji (2002) proposed that maintaining this taxon as a variety of *Cymbella turgidula* was not appropriate. They based their arguments on the co-occurrence of the nominate variety and the variety *nipponica*. Therefore, they proposed the name *Cymbella rheophila* T.Ohtsuka for this taxon at the specific rank.

Skvortsov and Noda (1971) described *Cymbella uenoi* Skvortsov, but did not indicate any type. According to Article 40.1 of the ICN, names of new genera or taxa of lower ranks published after 1958 are valid only when the type is indicated (McNeill et al. 2012), and therefore *Cymbella uenoi* is invalid. Tuji (2007), however, indicated a type for *Cymbella uenoi*, fulfilling the conditions required by the ICN. Therefore, the author of the name becomes *Cymbella uenoi* Skvortsov *ex* Tuji. Tuji (2007) also transferred Cymbella turgidula var. nipponica [≡ *Cymbella rheophila*] to that species, resulting in the name Cymbella uenoi f. nipponica, considering erroneously that the name *Cymbella uenoi* had priority under the name *Cymbella rheophila*.

Tuji (2007, p. 54) suggested the conspecificity of *Cymbella subturgidula* and *Cymbella uenoi*. The observations of the type material of *Cymbella uenoi* provided by Tuji (2007) and Cymbella uenoi f. nipponica [≡ *Cymbella rheophila*], compared with the type material of *Cymbella subturgidula* (i.e. morphometric characteristics), led us to agree with Tuji (2007). Since *Cymbella uenoi* was validated only in 2007, the valid names of this species are either *Cymbella rheophila* or *Cymbella subturgidula*, both published in 2002, and not *Cymbella uenoi* as stated by Tuji (2007). *Cymbella subturgidula* was described in The Diatoms of Europe, volume 3, published by Krammer on 28 January 2002 (Koeltz Scientific Books, pers. comm.), while *Cymbella rheophila* was not published before 29 July 2002, the date of acceptance of the paper. Therefore, the epithet *subturgidula* has priority over the epithet *rheophila*.

The re-analysis of the type material of *Cymbella subturgidula* allows us to broaden the metric data of this species compared to its original description. We observed a wider range of values for length (30.3–37.4 vs. 36–37 µm), breadth (9.0–12.8 vs. 10.0–11.0 µm), striae (9–11 vs. 10–11 in 10 µm) and punctae (21–24 vs. 24 in 10 µm) compared to Krammer’s (2002) description. Metric characteristics of the original material of Cymbella turgidula var. nipponica [≡ *Cymbella reophila*; ≡Cymbella uenoi f. nipponica, isolectotype designated here (Fig. 78)] and *Cymbella uenoi* (Table 1) also agree with the characteristics of the type population of *Cymbella subturgidula* (Skvortzov and Noda 1971, Ohtsuka and Tuji 2002, Tuji 2007).

Krammer (2002) described two isolated pores in *Cymbella subturgidula*. Similarly, Ohtsuka and Tuji (2002) and Tuji (2007) observed two isolated pores in the material of Cymbella turgidula var. nipponica [≡ *Cymbella rheophila*; ≡ Cymbella uenoi f. nipponica] and *Cymbella uenoi*. However, in the original sample of the lectotype of Cymbella turgidula var. nipponica we found some specimens with only one isolated pore (Fig. 88), and in recent material collected in Lake Biwa we found up to three isolated pores (Fig. 90).

**Figures 85–89.**
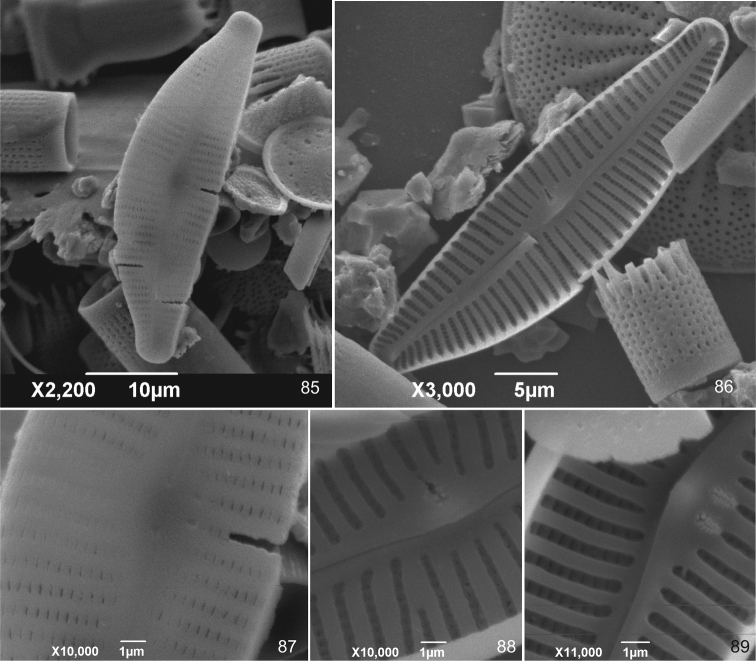
*Cymbella subturgidula* Krammer from Japan, in the original material of Cymbella tumidula var. nipponica Skvortsov [≡ *Cymbella rheophila* Ohtsuka], in sample 0983 **85, 87** External valvar view **86, 88, 89** Internal valvar view.

**Figures 90–97.**
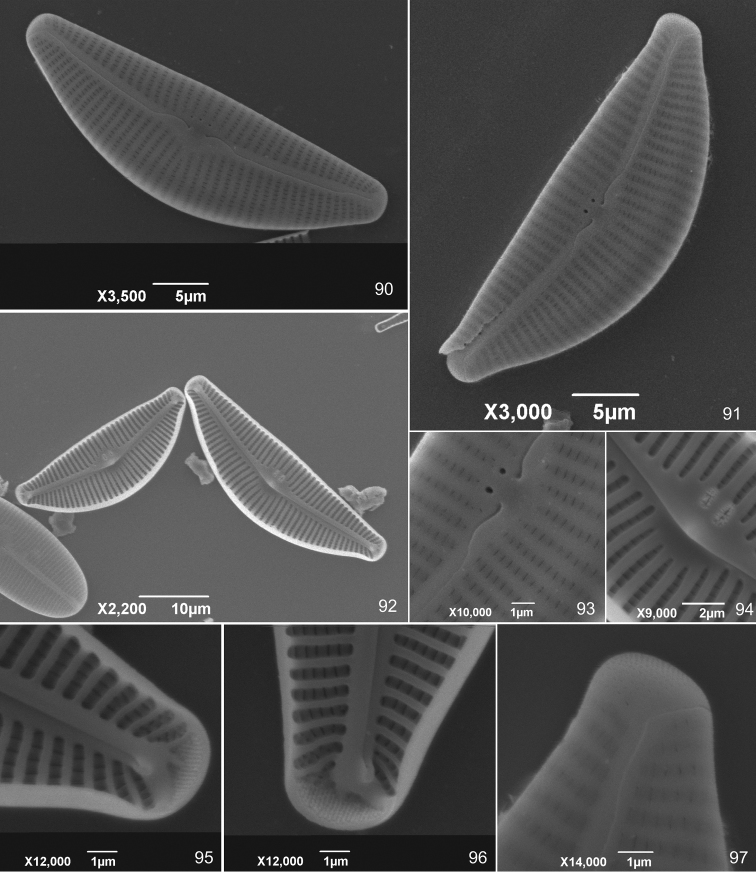
*Cymbella subturgidula* Krammer from Japan in sample 1093 **90, 91, 93, 97** External valvar view **92, 94, 95, 96** Internal valvar view.

## Discussion

Few studies have discussed the criteria to delimit infraspecific ranks in diatoms. Cox (1997) reviewed this issue and found contradictions among the criteria adopted by different researchers over time. In order to resolve this question, she proposed a pragmatic solution to delimit infraspecific taxonomic ranks. According to Cox (1997), the term variety should be used “for populations (within the same species) which are ecologically and morphologically distinct, in which there is no evidence of morphological intergrading under intermediate conditions”.

However, taxonomic analyses of some species complexes in diatoms using molecular data have demonstrated that Cox’s (1997) suggestions about the delimitation of varieties do not apply to every situation. *Sellaphora pupula* (Kütz.) Mereschk. sensu lato and *Nitzschia palea* (Kütz.) W.Sm. sensu lato, for example, have been shown to be an assemblage of pseudocryptic species that correspond, in the majority of instances, to populations with intergrading morphological characteristics (Mann et al. 2008; Trobajo et al. 2009). We have found a similar situation in populations that were clearly identified as *Cymbella tumida* (Bréb.) Van Heurck (unpublished data).

The criteria adopted by Krammer (2002, 2003) to delimit several specific and infraspecific taxa of *Cymbella* take into account variations between the type and similar individuals from other populations. It is important to note that, for Krammer (2002, 2003), types can be individuals marked or represented in some illustration, as well as a group of individuals mounted in a preparation, as defined in the ICN (McNeill et al. 2012).

Because of the lack of taxonomic studies on the *Cymbella affinis*/*tumidula*/*turgidula* species complex using molecular data, we opted to use Krammer’s concept in order to attempt to organize this confusing group. This criterion is usual as a reference to circumscribe groups with similar morphologies, even if individuals with these morphologies sometimes overlap. Some authors prefer to define similar groups as “morphodemes”, which have no nomenclatural status. However, in some cases, this definition sounds more similar to the old taxonomy when any taxonomic unit could be denominated by different names, but scientifically by a long sentence that is more similar to the current “diagnosis”. Although the concept of varieties used here implies the publication of nomenclatural novelties, none of them are new proposals, but rather are simply adjustments of already existing names that are in confusing combinations, because of a misinterpretation by Krammer (2002) as well as the history of the taxa and the evolution of nomenclatural rules.

The criteria of delimitation of taxa and the weight of characters in diatoms are variable from group to group (Mann 1999). While in some groups the density of striae is a good morphological indicator of different species, in other groups this can be irrelevant (Abarca et al. 2014). Different sizes and valve outlines are not always good characters to delimitate taxa such as in the complex *Encyonema silesiacum*/*minutum*/*ventricosum* (unpublished data). Thus, it is common to find taxa with polythetic definitions, that is, in which a set of characteristics, sometimes interweaving with other taxa, are taken into account (Needham 1975). The delimitation of *Cymbella* taxa is a clear example of polythethism (Krammer 2002).

In this context, several characters must be considered in their characterization and identification of taxa. The degree of dorsiventrality of the valve, for example, is slightly higher in *Cymbella affinis* than in *Cymbella tumidula*, and even higher in *Cymbella schilleri* Krammer (2002, Fig. 26: 7, 8) than in *Cymbella orientalis* Lee (Krammer 2002, Fig. 26: 1–6). Another example is the thickness of the striae. The striae of *Cymbella tumidula* are narrower than in individuals of *Cymbella affinis* in LM, even in specimens of the same size. This is often the result of thick intercostae and the size of areolae, which can only be observed in SEM (Krammer 2002, Figs 5: 1, 23: 18). Thus, these characters, alone, seem to be unimportant but in combination with further features can provide a better concept of the taxa.

Ultrastrutural characterizations by SEM are also important in diatoms, but not always possible using the original material. Records from the literature have demonstrated that representatives of the *Cymbella affinis*/*tumidula*/*turgidula* species complex present similar internal ultrastructure of the isolated pores (i.e., aperture covered by small teeth) and the intercostae. This is the case of *Cymbella affinis* (=*Cymbella excisa* sensu Krammer 2002, Figs 5: 1), Cymbella affinis var. subcapitata (Krammer 2002, Figs 10: 16–18) Cymbella tumidula var. tumidula (=*Cymbella affinis* sensu Krammer 2002, Fig. 23: 18), and *Cymbella subturgidula* (Figs 89, 94), which are similar to *Cymbella cymbiformis* C.Agardh, type of the genus (Krammer 2002, Figs 5: 2, 3). However, the internal structure of isolated pores and the intercostae of these taxa are different to other species such as *Cymbella aspera* (Ehrenb.) Perag. (Krammer 2002, Fig. 5: 4), *Cymbella neolanceolata* W.Silva (=*Cymbella lanceolata* sensu Krammer 2002, Fig. 5:5), *Cymbella neocistula* Krammer (2002, Figs 5: 6, 7). This demonstrates that the complex *Cymbella affinis*/*tumidula*/*turgidula* is morphologically closer related to the type of the genus, than to the other species of the same genus.

The economic and ecological uses of diatoms require a refined taxonomy, which is more detailed than simply species complexes. This is especially true for bioassessments using diatoms (European Committee for Standardization 2003). *Gomphonema lagenula* Kütz. has for a long time been treated as synonym of *Gomphonema parvulum* Kütz., since the variability in the shape of the ends were considered insufficient to distinguish the taxa (VanLandingham 1971). However, differences in the ecological preferences of these two taxa were recorded and the independence of the two species has been confirmed by molecular data (Kermarrec et al. 2013, Abarca et al. 2014). Differences between the ecological preferences of Nitzschia palea (Kütz.) W.Sm. var. palea and Nitzschia palea var. debilis (Kütz.) Grunow have also been recorded, and simply morphological characters are insufficient to separate the series of phenotypic expressions subscribed to *Nitzschia palea* (Trobajo et al. 2009). Thus, the efficiency in the use of diatoms in activities such as bioassessments needs accuracy and taxonomic harmonization (Manoylov 2014). But this will only be possible if important characteristics such as ecological preferences can be permanently attached to a taxon or an accessible designation, which is facilitated by the establishment of correct types and by the knowledge of these types.

Naturally, beside the knowledge of types, supplementary studies are necessary to record phenotypic plasticity resulting from different ecological conditions or by life cycles. Such studies should be carried out in natural or cultivated samples in order to observe a more realistic concept of the species (Mann 1999). These studies will allow us to observe slight changes in the morphology of the valves (i.e., outline, measures, etc.) such as some of those observed in the type population of the taxa discussed here. Moreover, these studies can possibly verify the relationship among morphological characters, which are associated in a polythetic way during the establishment of specific concepts and in their use in the identification of taxa of similar groups.

## Conclusion

The process of lectotypification can markedly influence the identity of some taxa and can sometimes substantially change the relation to other taxa. The designation of a type for *Cymbella affinis* resulted in a profound restructuring of *Cymbella affinis*, *Cymbella excisa* and *Cymbella tumidula*. *Cymbella excisa* has been shown to be the same taxon at the species level as *Cymbella affinis*, but because of its specific morphology is treated herein at a different rank. The epithet *affinis* has priority over the epithet *excisa*, as defined by the criterion of the first effective publication. Thus, four infraspecific taxa of *Cymbella excisa* were transferred to *Cymbella affinis*.

The lectotypification of *Cymbella tumidula* Grunow and comparisons with the lectotype of *Cymbella affinis* allowed us to conclude that the two species are independent. Cymbella affinis var. procera was treated as a new species, which is closer to *Cymbella tumidula* than *Cymbella affinis* because of morphological similarities. Infraspecific taxa described by Krammer (2002) within *Cymbella affinis* had small differences in relation to the type of *Cymbella tumidula*, and they are recombined herein.

The analysis of the type and the history of taxa such as *Cymbella subturgidula* Krammer, *Cymbella rheophila* Ohtsuka, and *Cymbella uenoi* Skvortsov *ex* Tuji allowed us to conclude that these taxa are conspecific, and to determine that the epithet *subturgidula* has priority.

## Supplementary Material

XML Treatment for Cymbella affinis

XML Treatment for Cymbella affinis var. excisa

XML Treatment for Cymbella affinis var. neoprocera

XML Treatment for Cymbella affinis var. angusta

XML Treatment for Cymbella affinis var. subcapitata

XML Treatment for Cymbella tumidula Grunow var. tumidula

XML Treatment for Cymbella tumidula var. procera

XML Treatment for Cymbella tumidula var. salinarum

XML Treatment for Cymbella turgidula

XML Treatment for Cymbella tropica

XML Treatment for Cymbella subturgidula
